# Beneficial effects of seaweed-derived components on metabolic syndrome via gut microbiota modulation

**DOI:** 10.3389/fnut.2023.1173225

**Published:** 2023-06-15

**Authors:** Liqing Zang, Maedeh Baharlooeian, Masahiro Terasawa, Yasuhito Shimada, Norihiro Nishimura

**Affiliations:** ^1^Graduate School of Regional Innovation Studies, Mie University, Tsu, Mie, Japan; ^2^Mie University Zebrafish Research Center, Mie University, Tsu, Mie, Japan; ^3^Department of Marine Biology, Faculty of Marine Science and Oceanography, Khorramshahr University of Marine Science and Technology, Khorramshahr, Iran; ^4^Konan Chemical Manufacturing Co., Ltd., Yokkaichi, Mie, Japan; ^5^Department of Integrative Pharmacology, Mie University Graduate School of Medicine, Tsu, Mie, Japan; ^6^Department of Bioinformatics, Mie University Advanced Science Research Promotion Center, Tsu, Mie, Japan

**Keywords:** seaweed, gut microbiota, short-chain fatty acids, obesity, diabetes, metabolic syndrome

## Abstract

Metabolic syndrome comprises a group of conditions that collectively increase the risk of abdominal obesity, diabetes, atherosclerosis, cardiovascular diseases, and cancer. Gut microbiota is involved in the pathogenesis of metabolic syndrome, and microbial diversity and function are strongly affected by diet. In recent years, epidemiological evidence has shown that the dietary intake of seaweed can prevent metabolic syndrome via gut microbiota modulation. In this review, we summarize the current *in vivo* studies that have reported the prevention and treatment of metabolic syndrome via seaweed-derived components by regulating the gut microbiota and the production of short-chain fatty acids. Among the surveyed related articles, animal studies revealed that these bioactive components mainly modulate the gut microbiota by reversing the Firmicutes/Bacteroidetes ratio, increasing the relative abundance of beneficial bacteria, such as *Bacteroides*, *Akkermansia*, *Lactobacillus*, or decreasing the abundance of harmful bacteria, such as *Lachnospiraceae*, *Desulfovibrio*, *Lachnoclostridium*. The regulated microbiota is thought to affect host health by improving gut barrier functions, reducing LPS-induced inflammation or oxidative stress, and increasing bile acid production. Furthermore, these compounds increase the production of short-chain fatty acids and influence glucose and lipid metabolism. Thus, the interaction between the gut microbiota and seaweed-derived bioactive components plays a critical regulatory role in human health, and these compounds have the potential to be used for drug development. However, further animal studies and human clinical trials are required to confirm the functional roles and mechanisms of these components in balancing the gut microbiota and managing host health.

## 1. Introduction

Metabolic syndrome comprises a cluster of metabolic disorders associated with abdominal obesity, diabetes, hypertension, hyperlipidemia, hyperglycemia, atherosclerosis, cancer, and cardiovascular disease (1). The increasing prevalence of metabolic syndrome worldwide is developing into a severe health problem and economic burden. The bulk of modern lifestyles, such as increased high-caloric intake and decreased physical activity, result in a high incidence of metabolic syndrome. Patients with metabolic syndrome should first be consulted for lifestyle modifications to reduce the risk of developing metabolic syndrome, in the light of which, diet and exercise modifications, weight loss, and smoking cessation have been emphasized. However, once lifestyle interventions fail to control disease progression, drug therapy is required to direct the risk factors. Current drug therapies for metabolic syndrome mainly focus on treating dysglycemia, dyslipidemia, and hypertension separately. However, several adverse side effects of these medications have been reported, including depressed mood and anxiety (2). Therefore, the interest in natural products as potential treatments with minimal side effects has increased. Instead of calorie or single-nutrient restrictions, functional food-based dietary interventions are recommended to prevent or treat metabolic syndrome. Seaweeds (also known as macroalgae) and their bioactive components have recently attracted attention as potential functional foods for managing metabolic syndrome in humans (3).

Seaweeds comprise more than 25,000 species (4) and are essential for marine ecosystems. They are crucial blue carbon sinks for a sustainable economy, and their wide variety, abundance, and fast proliferation make them attractive future bioresources (5). Edible seaweeds have been consumed as vegetables in East Asian countries for several thousand years, and their ability to improve human health has recently been recognized in the Western world (6). Seaweeds can be classified into three major groups based on the thallus pigment color: brown (*Phaeophyceae*), red (*Rhodophyta*), and green (*Chlorophyta*) (6, 7). All groups are rich in multiple valuable macro-and micronutrients, including carbohydrates, proteins, polyunsaturated fatty acids (PUFAs), dietary fiber, vitamins, minerals, and bioactive compounds (8, 9). Polysaccharides are the major components of seaweed biomass, and they account for up to 76% of the dry weight of certain species (10). Phenolic compounds and proteins derived from seaweeds have been widely studied as potential functional compounds associated with antioxidant, antibacterial, antiviral, and antifungal properties (11). Therefore, seaweeds are considered aquatic plant-based proteins for sustainable nutrition (12) and can provide a potential source of probiotics (13, 14).

The human body contains at least 100 trillion microbial cells, of which 95% are harbored by the human gastrointestinal tract (15). Microorganisms that inhabit the human gut include bacteria, viruses, archaea, fungi, and protozoa, collectively termed the gut microbiota (16). The gut microbiota compiles complex ecosystems in which microorganisms interact with both each other and the host, thereby exerting a profound influence on human health and physiology (17, 18). The gut microbiota can be considered a “microbial organ” that contributes to host physiological processes, including energy harvesting and storage, metabolism (such as the fermentation of dietary fiber or host-derived glycans) (19), the management of the stability of the human intestinal microenvironment (20), and is closely related to the development and regulation of the host immune system (21). Additionally, short-chain fatty acids (SCFAs), the main end products of gut microbial fermentation of indigestible foods, are crucial for intestinal health and are involved in crosstalk between the gut and peripheral tissues (22, 23). Furthermore, key metabolites produced by the gut microbiota play critical roles and interact with several vital metabolic pathways related to incretin production, insulin signaling, and inflammation (24). In recent years, it has been demonstrated that dysbiosis of the gut microbiota, which indicates an alteration in its composition, is closely associated with the development of several diseases (25), such as metabolic syndrome (26–28), inflammatory bowel disease (29–32), and central nervous system-related disorders (33–35).

In this review, we provide an up-to-date summary of the current state of knowledge regarding bioactive compounds derived from seaweeds and how these compounds prevent or treat metabolic syndrome and related diseases by modulating the gut microbiota. Finally, future perspectives on seaweed and gut microbiota are discussed.

## 2. Methodology

**Figure 1:**
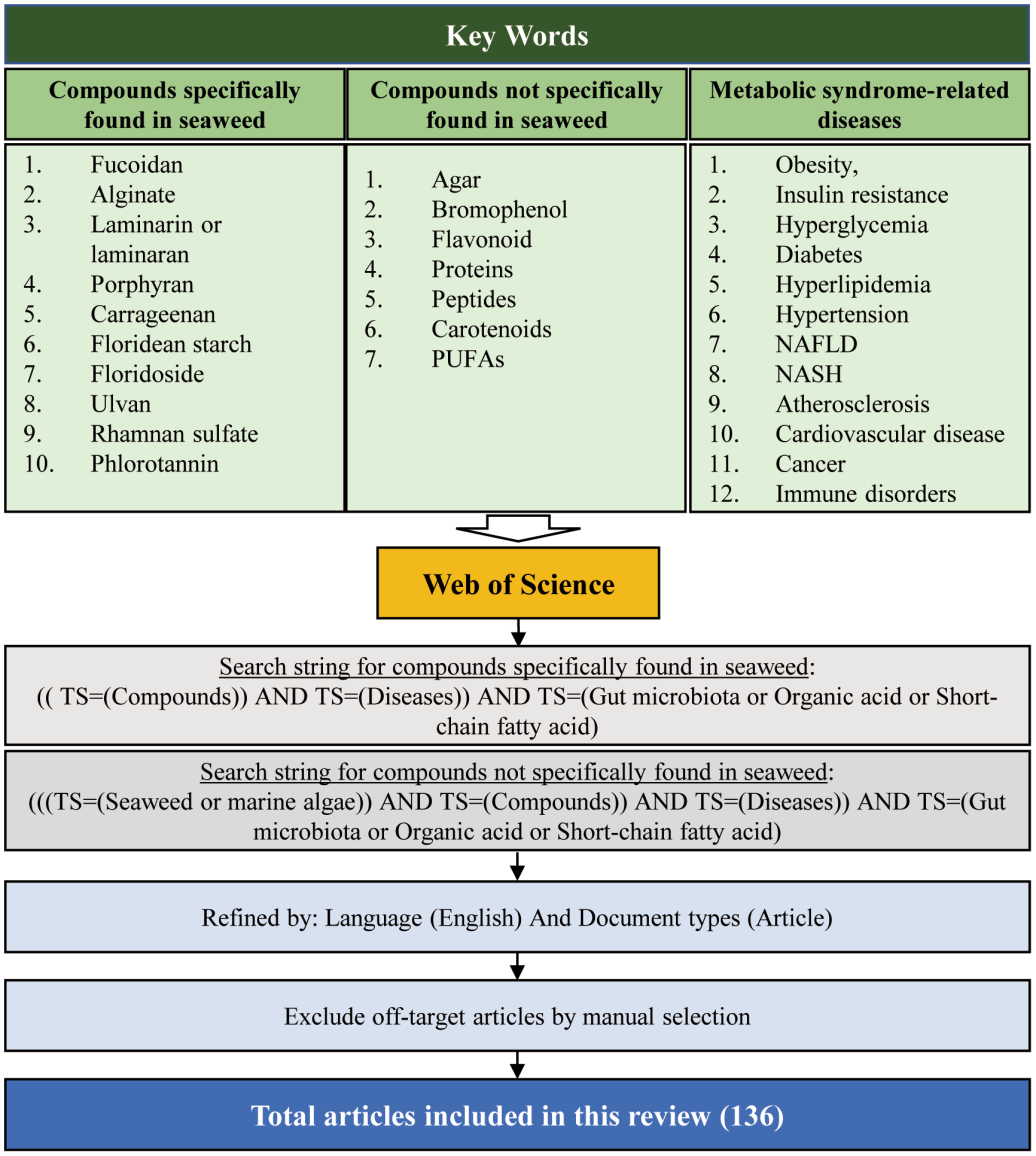
Overview of the article selection process for this review.

Systemic literature was obtained through an advanced search of Web of Science interfaces (last accessed on June 10, 2022). The detailed steps of the literature search are shown in Figure 1. Peer-reviewed scientific articles were collected using the following keywords: “Fucoidan, “Alginate,” “Laminarin” or “Laminaran,” “Agar,” “Porphyran,” “Carrageenan,” “Floridean starch,” “Floridoside,” “Ulvan,” “Rhamnan sulfate,” “Bromophenol,” “Phlorotannin,” “Flavonoid,” “Proteins,” “Peptides,” “Carotenoids,” “PUFAs,” “Obesity,” “Insulin resistance,” “Hyperglycemia,” “Diabetes,” “Hyperlipidemia,” “Hypertension,” “Non-alcoholic fatty liver disease (NAFLD),” “Non-alcoholic steatohepatitis (NASH),” “Atherosclerosis,” “Cardiovascular disease,” “Cancer,” “Immune disorders,” “Gut microbiota,” “Organic acid,” and “Short-chain fatty acid.” For compounds not specifically found in seaweed, such as “Agar,” “Bromophenol,” “Flavonoid,” “Peptides,” “Proteins,” “Carotenoids,” and “PUFAs” were searched simultaneously with “seaweed” or “marine algae.” In addition, purified bioactive fractions were not included in the search. Only research articles published in English were included.

**Figure 2:**
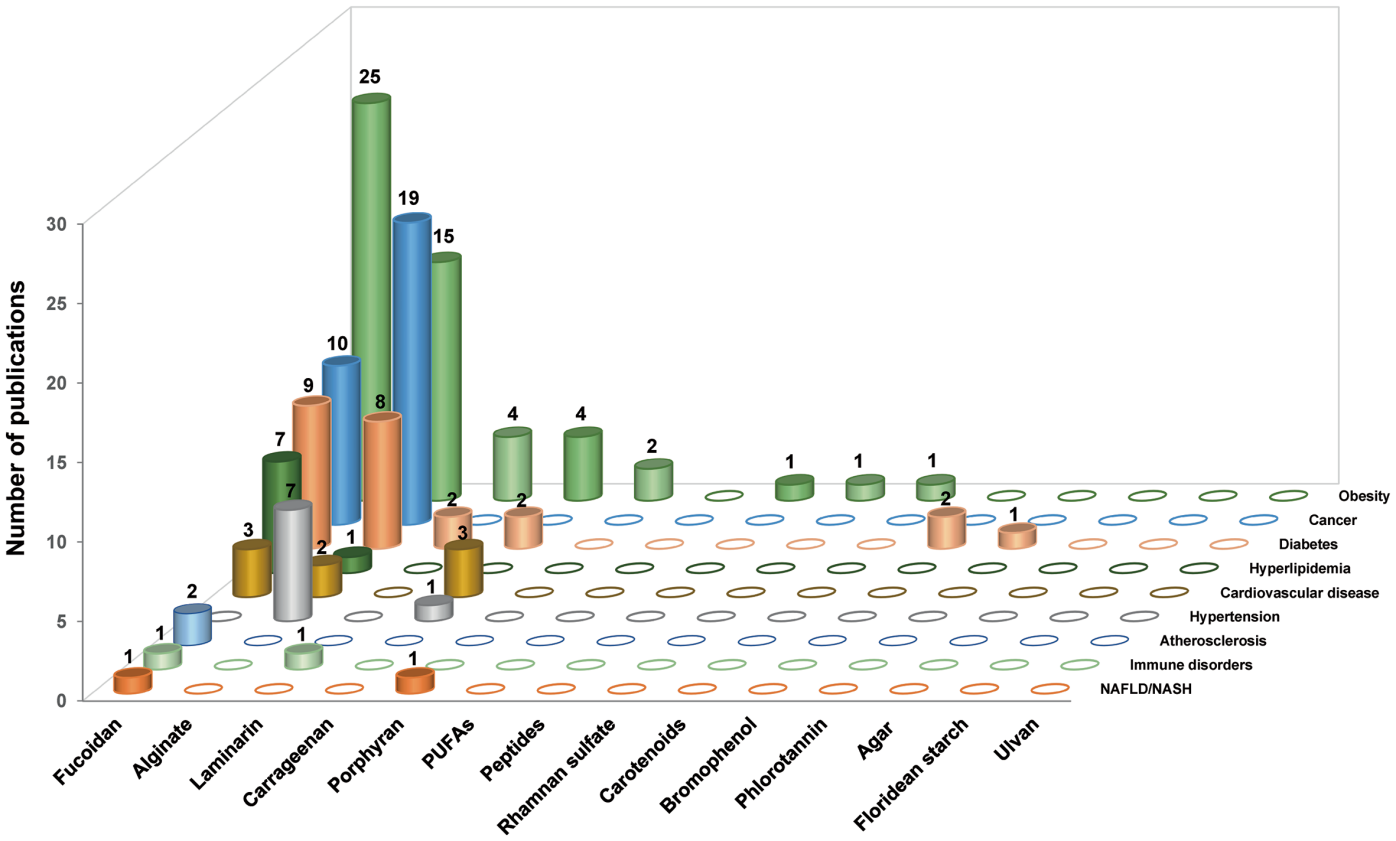
The number of research publications based on the relationship between gut microbiota, different seaweed-derived components, and different types of metabolic syndrome-related diseases. A total of 136 articles were sourced.

Initially, we collected papers using the plural forms of different words, such as the names of 17 “seaweed or marine algae-derived compounds” combined with 12 “metabolic syndrome-related diseases,” respectively. Compounds were ranked sequentially based on the number of relevant publications for each disease (Supplementary Figure S1). Furthermore, 136 papers were collected when filtered using a combination of three factors: the “compound’s name” AND “metabolic syndrome-related diseases’ name” AND “gut microbiota or organic acid or SCFAs” (Figure 2). Fucoidan was found to be the most prevalent seaweed-derived component investigated, with 58 articles revealing its potential ability to prevent or treat metabolic syndrome-related diseases by modulating the gut microbiota. Among the 12 diseases searched for, the highest number of publications (25) related to “obesity,” and no published reports for “hypertension” included the relationship between fucoidan and gut microbiota. Alginate is also a well-studied compound and 52 articles were found pertaining to it. However, articles on “NAFLD/NASH,” “Immune disease,” and “Atherosclerosis” that included the relationship between alginate and gut microbiota have yet to be published. There were comparatively few related publications on carrageenan (*n* = 12), laminarin (*n* = 7), porphyran (*n* = 3), bromophenol (*n* = 2), peptides (*n* = 1), rhamnan sulfate (*n* = 1), carotenoids (*n* = 1), and phlorotannin (*n* = 1). Notably, most of the articles included in this study were published within the last 5 years, indicating that the health benefits of seaweed-derived components via regulation of the gut microbiota have recently attracted research attention. In addition, this study found no reports on seaweed-derived PUFAs, agar, floridean starch or floridoside, and ulvan; therefore, future studies are required. This section briefly summarizes the representative *in vivo* studies of seaweed-derived components that affect metabolic syndrome-related diseases via gut microbiota modulation.

## 3. Seaweed components

Seaweeds contain nutritional elements such as carbohydrates, proteins, lipids, vitamins, and minerals, and the proportional contents of the elements derived depend on the habitat of the particular seaweed, the season in which it was harvested (6), and the extraction and purification methods employed (36, 37). The major bioactive compounds in seaweeds that are beneficial to human health comprise polysaccharides, polyphenols, proteins, peptides, and phytochemicals. In this section, we briefly summarize representative components that promote human health and/or ameliorate diseases. Details of the major seaweed-derived bioactive compounds, including seaweed source, characteristics, and biological activities, are listed in Table 1.

**Table 1:** Characteristics and biological activities of seaweed-derived bioactive compounds.

Seaweed-derived bioactive components		Primary seaweed source	Characteristics	Biological activity
Polysaccharides	Fucoidan	Brown seaweed (*Saccharina japonica*, *Ascophyllum nodosum*, etc.)	Formed by mannose, galactose, glucose, xylose, uronic acids, acetyl groups, and proteins	Anticancer, antidiabetic, antihyperlipidemia, antioxidant, anti-inflammatory, anticoagulant, antithrombotic, antiviral activities	Alginate	Brown seaweed (*Laminaria japonica*, *Ascophyllum nodosum*, etc.)	Water-soluble linear polysaccharide molecule containing linear copolymers	Anti-cardiovascular disease, anti-inflammatory, antitumor, antioxidant activities	Laminarin	Brown seaweed (*Laminaria japonica*, *Saccharina longicruris*, etc.)	β-(1 → 3)-glucan containing β-(1 → 6)-linked branches	Anticancer, anti-metastatic, antioxidant activities	Agar	Red seaweed (*Gracilaria* sp. etc.)	Galactose-based heterogeneous polysaccharide, composed of 70% agarose and 30% agaropectin polymers	Anti-pathogen, cellular ionic equilibrium maintenance, protection from bad conditions	Porphyran	Red seaweed (*Porphyra* sp. etc.)	Consists of a linear skeleton of alternating 1,3-linked β-D-galactosyl units (G) and 1,4-linked L-residues	Antioxidant, anti-inflammatory, immunomodulatory, anticancer, anti-aging, anti-allergenic, anti-hyperglycemic, anti-hyperlipidemic properties	Carrageenan	Red seaweed (*Agardhiella sp., Chondrus crispus,* etc.)	Formed by alternate units of d-galactose and 3,6-anhydro-galactose (3,6-AG) linked together by α-1,3 and β-1,4-glycosidic bonds	Antiviral, antitumor, antiendotoxic, immunomodulator activities	Floridean starch Floridoside	Red seaweed (*Gracilaria* spp., etc.)	Semi-crystalline polysaccharide Natural galactosyl glycerol	Antioxidant activity	Rhamnan sulfate	Green seaweed (*Monostroma* sp. etc.)	Composed of octa-saccharide repeating units with a linear chain of α-1,3-linked l-rhamnose attached to α-1,2-linked branched chains	Antioxidative, anticoagulant, anti-inflammatory, antitumor, antiviral activities	Ulvan	Green seaweed (*Ulva* sp.)	Composed of l-rhamnose, d-xylose, d-glucose, iduronic acid, and d-glucuronic acid	Anti-inflammatory, antioxidant, antibacterial, antiviral, antiherpetic, anticancer activities
Polyphenols	Phlorotannins	Brown seaweed (*Sargassum vulgare*, *Fucus vesiculosus*, etc.)	Made up of polymeric chains of base phloroglucinol residues	Neuroprotective, antidiabetic, anticancer, antioxidant, anti-inflammatory, antihypertensive, hepatoprotective, antimicrobial activities	Bromophenols	Red seaweed (*Rhodomela larix*, etc.)	Secondary metabolites biosynthesized by bromoperoxidases, bromases, laccase, hydrogen peroxide, and bromide	Antioxidant, anti-radical, anticancer, antimicrobial, anti-diabetic, anti-obesity, and anti-inflammatory properties	Flavonoids	Brown, red, and green seaweeds	Hydroxylated polyphenolic compounds	Anti-allergenic property
Phytochemicals	Carotenoid	Brown, red, and green seaweeds	Terpenoid pigments with a linear C40 polyene chain	Antioxidant, anticancer, anti-inflammatory activities	Polyunsaturated fatty acids	Brown, red, and green seaweeds	Fatty acids with more than two double bonds in their backbones	Anti-cardiovascular diseases, anticancer, anti-diabetes activities
Proteins	Lectins	Brown, red, and green seaweeds	Glycoproteins with a non-immune origin that bind to carbohydrates	Antibacterial, anti-inflammatory, antiviral, anticancer activities	Phycobiliprotein	Brown, red, and green seaweeds	Major light-harvesting pigments in red seaweeds	Fluorescence property
Peptides		Brown, red, and green seaweeds	Composed of approximately 3–40 amino acids	Antioxidant, anticancer, antihypertensive, antiatherosclerosis activities

### 3.1. Polysaccharides

In seaweed, polysaccharides and polycarbohydrates are biomacromolecules composed of repeating monosaccharide units linked by glycosidic bonds. The relevant taxonomies of polysaccharides differ between seaweeds: fucoidans, alginates, and laminarins are predominantly found in brown seaweeds; agars, porphyrins, carrageenan, and floridean starch in red seaweeds; and rhamnan sulfate and ulvan are found in green seaweeds (38, 39).

Fucoidans comprise a family of polymeric molecules derived from sulfated polysaccharides consisting of l-fucose (6-deoxy-l-galactose) (40). They are primarily produced by brown algae and, to a lesser extent, by other marine aquatics such as sea cucumbers (41). Fucoidan provides an outer cell wall structure and a hydrophilic coating to prevent seaweed from drying during low tides (42). In general, fucoidans have long, simple structures based on their fucose and sulfate groups. Fucoidans are usually formed by monosaccharides, such as mannose, galactose, glucose, xylose, uronic acids, acetyl groups, and proteins (43), and some fucoidans contain monosaccharides with alternating α (1 → 3) and α (1 → 4) bonds (44). Over the past several decades, fucoidan has been shown to have anticancer, antidiabetic, anti-hyperlipidemic, antioxidant, anti-inflammatory, anticoagulant, antithrombotic, and antiviral activities (45). Recently, the prebiotic effects of fucoidans have gained attention in both *in vitro* and *in vivo* studies (46).

Alginate is a water-soluble linear polysaccharide containing linear copolymers composed of blocks of (1,4)-linked β-D-mannuronate (M) and-l-guluronate (G) (47). Alginate is the most abundant polysaccharide naturally present in the cell walls of brown seaweeds (48) and is a naturally occurring anionic polymer. They have a variety of biochemical and biomedical applications, such as wound healing, drug delivery, cell culture, and tissue engineering (blood vessels, bones, cartilage, muscles, nerves, pancreas, and liver), owing to their biocompatibility, low toxicity, low cost, and mild gelation caused by the addition of divalent cations such as Ca^2+^ (49, 50). Hydrogels are composed of hydrophilic polymers with a high water content and three-dimensional cross-linked networks. Depolymerized alginate, also known as alginate oligosaccharide, is widely used in the food and pharmaceutical industries. Alginate oligosaccharides have received attention for their biological functions, such as decreasing the risk of cardiovascular disease in addition to having anti-inflammatory, antitumor, and antioxidant activities (51).

Laminarin, or laminaran, is a type of β-(1 → 3)-glucan containing β-(1 → 6)-linked branches (52). It is mainly found as a storage polysaccharide (up to 35% of the dry weight) in brown algae. In Asian countries, laminarin is formulated for use as a food, medicine, and dietary supplement (53), and it provides a wide range of bioactivities with anticancer, anti-metastatic, and antioxidant properties (46), making it increasingly attractive as an essential nutritious source or functional food.

Agar is a galactose-based, heterogeneous polysaccharide derived from the cell walls of red algae. It is a thermo-reversible gelling agent composed of 70% agarose and 30% agaropectin polymers. Agarose (or agaran) is a gel-forming component with a linear chain of 3-O-substituted β-d-galactopyranosyl units linked by (1 → 4) chains to 3,6-anhydro-α-l-galactopyranosyl units (54). Agaropectin is a branched, non-gelling component of agar that contains the D- and L-isomers of galactose. Agar is best known as a growth medium to identify and enumerate microorganisms (55). The properties of agar include anti-pathogenic activity (56), maintenance of cellular ionic equilibrium (57), and protection from extreme salinity, pH, temperature, and desiccation (58). Agars are used as phycocolloids in diverse industries such as food, pharmaceuticals, cosmetics, medicine, and biotechnology (59).

Porphyran is a water-soluble polysaccharide within the algal cell wall and intercellular space and is the main component of porphyrin. A typical porphyran structure consists of a linear skeleton of alternating 1,3-linked β-D-galactosyl units (G) and 1,4-linked L-residues (60). The key roles of porphyrans in functional foods, cosmetics, and pharmaceuticals are well established. This polysaccharide exhibits many biological properties, including antioxidant, anti-inflammatory, immunomodulatory, anticancer, anti-aging, anti-allergenic, anti-hyperglycemic, and anti-hyperlipidemic effects (61).

Carrageenans are linear hydrophilic sulfated polysaccharides of the galactan family extracted from various genera of red algae. Carrageenans contain 15–40% ester sulfate, and they are formed by alternating units of d-galactose and 3,6-anhydro-galactose (3,6-AG) linked together by α-1,3 and β-1,4-glycosidic bonds. Carrageenans are classified into λ-, κ-, ι, β, μ-, ν-, and θ-carrageenan, depending on the repeating disaccharide units, all of which contain 22–35% sulfate groups. Due to their fundamental structure, carrageenans exhibit various physicochemical properties and are used for gelling, solubility, electronegativity, and synergistic effects (62). Gel formation from carrageenan is extensively used in the food, pharmaceutical, and bioengineering industries (63). Carrageenans have been reported to exhibit antiviral (64), anti-tumor (65), anti-endotoxic (66), and immunomodulatory activities (67).

Floridean starch and floridoside are the main storage carbohydrates of red algae. Floridean starch comprises a semi-crystalline polysaccharide that is similar to starch, but in green algae, differing from land plants due to its amylopectin-like glucan and amylose contents (68). In addition to serving as an effective photosynthetic substrate for red algae, floridean starch provides an efficient method for producing hydroxymethylfurfural (69). Floridoside (α-d-galactopyranosyl-(1,2)-glycerol), a natural galactosyl glycerol, is the main soluble photosynthetic molecule synthesized in the cytoplasm of red algae and has antioxidant properties (70).

Rhamnan sulfate (RS) and ulvans are sulfated polysaccharides extracted from green algae. They are sometimes categorized into the same group; however, there are clear differences between them with respect to their sugar compositions and main-chain structures (71). RS is obtained from the cell walls of the green alga *Monostroma* sp. and contains varied amounts of l-rhamnose and d-glucose, respectively (72). RS is composed of octa-saccharide repeating units with a linear chain of *α*-1,3-linked l-rhamnose attached to *α-*1,2-linked branched chains, to which several units are bound to macromolecular polysaccharides with molecular weights ranging from tens of thousands to millions (71). RS has therapeutic effects in metabolic syndrome-related disorders and provides antioxidative, anticoagulant, anti-inflammatory, and antitumor benefits (73, 74). Furthermore, the RS from *M. nitidum* has antiviral effects against several viruses (75), especially the wild-type SARS-CoV-2 and the delta variant (76). Ulvan is mainly obtained from *Ulva* sp., and it is primarily composed of l-rhamnose, d-xylose, d-glucose, iduronic acid, and d-glucuronic acid (77). Similar to RS, ulvan is also a potential candidate with anti-inflammatory, antioxidant, antibacterial, antiviral, antiherpetic, anticancer, biomedical, and pharmacological activities (78).

### 3.2. Polyphenols

Polyphenols are a group of heterogeneous compounds that contain a multitude of phenolic structures, ranging from simple molecules to highly polymerized compounds, where each phenolic compound contains one or more hydroxyl groups. Seaweeds have been found to contain several polyphenolic compounds (5–30% of the dry algal mass), including catechins, flavonols, and phlorotannins. Green and red algae contain the highest percentages of phenolic compounds such as phenolic acids, flavonoids, and bromophenols, whereas brown algae have the highest concentration of phlorotannin polyphenols (79). The three main types of chemically diverse polyphenols found in seaweeds are phlorotannins, bromophenols, and flavonoids.

Phlorotannins are polyphenolic derivatives found primarily in brown seaweeds (5–12% of the dry algal mass), and they are structurally distinct from tannins produced by terrestrial plants. These compounds are composed of polymeric chains of basic phloroglucinol residues (1,3,5-trihydroxybenzene) connected by C–C or C–O–C interactions. Phlorotannins have been found to have neuroprotective, antidiabetic, anticancer, antioxidant, anti-inflammatory, antihypertensive, hepatoprotective, and antimicrobial properties (80).

Bromophenols (BPs) are polyphenolic compounds with one or more benzene rings, hydroxyl substituents, bromine, and other groups in their structure (81). Marine BPs are conventional secondary metabolites biosynthesized by bromoperoxidases, bromases, laccases, hydrogen peroxide, and bromide (82). BPs exhibit diverse biological activities, including antioxidant (83), antiradical (84), anticancer (85), antimicrobial (86), anti-diabetic (87), anti-obesity (88), and anti-inflammatory properties (89).

Flavonoids are hydroxylated polyphenolic compounds with various structures that are found as aglycones or glycosides in many fruits and vegetables. Seaweeds such as *Ulva clathrata* also have high flavonoid content (90). With respect to their chemical structure, flavonoids consist of 15 carbons and include phenyl-benzo-γ-pyrans (C6-C3-C6), known as nucleus flava, comprising two phenyl rings (A and B) linked by a heterocyclic ring C (pyran). Flavonoids in brown algae have been reported to exhibit anti-allergenic activities (91).

### 3.3. Phytochemicals

Phytochemicals are plant-based chemicals produced via both primary and secondary metabolic pathways. Phytochemicals serve as antioxidant compounds but are not considered essential nutrients. There exists evidence of their beneficial health effects, such as those pertaining to carotenoids and PUFAs.

Carotenoids are terpenoid pigments that contain linear C40 polyene chains. They are abundant in seaweeds, are generally found in chloroplasts, and function in photosynthesis (92). Beta-carotene (β-carotene), lutein, zeaxanthin, astaxanthin, and fucoxanthin are essential carotenoids. Among these, β-carotene is one of the most abundant carotenoids in seaweeds and is a primary source of vitamin A. The powerful antioxidant properties of β-carotene make it widely used in photoprotection (93). Zeaxanthin is a dihydroxy-form derivative of β-carotene, whereas lutein is a dihydroxy-form derivative of α-carotene. In addition to being major components of macular pigments in the retina (eyesight), lutein and zeaxanthin are essential for eyesight and the prevention of strokes and lung cancer (94). Astaxanthin is a red fat-soluble pigment without pro-vitamin A properties, but has been shown to have antioxidant, anti-inflammatory, and immune-enhancing biological activities in humans and animals (95, 96). Fucoxanthins are among the most abundant carotenoids in seaweeds and are mainly found in brown seaweeds and microalgae. Owing to its potent health benefits, fucoxanthin has been the focus of much attention due to its ability to prevent cancers through its antioxidant activity and apoptosis-inducing action (97).

Polyunsaturated fatty acids (PUFAs) are fatty acids with more than two double bonds in their backbones. Many brown seaweeds possess high levels of total lipids in their dry weights (ranging from 1 to 10% per dry weight). These seaweeds are an immense source of omega-3 PUFAs (such as EPA and stearidonic acid [18,4*n*–3)], whereas omega-6 PUFAs are predominantly arachidonic acid (ARA, 20:4*n*–6) (98). These nutrients play crucial roles in brain development, cognition, prevention of neurodegeneration, regulation of inflammatory and immune responses, and prevention of many diseases, including cardiovascular diseases, cancer, and diabetes (99).

### 3.4. Proteins derived from seaweeds

Seaweed proteins contain several amino acids, including glycine, arginine, alanine, and glutamic acid. The protein content varies from 10 to 40% per dry weight, depending on the species and season. In general, the content was low for brown seaweeds (3% ± 15% per dry weight), moderate for green algae (9% ± 26% per dry weight), and high for red seaweeds (with a maximum of almost 50% of dry weight). The two functionally active proteins in seaweeds are lectins and phycobiliproteins (8). Lectins are glycoproteins of non-immune origin that bind to carbohydrates and are associated with many biological processes, such as intercellular communication and red blood cell agglutination (100). They have been detected in several seaweed species and possess antibacterial (101), anti-inflammatory (102), antiviral, and anticancer properties (103). The phycobiliprotein family of fluorescent proteins found in red seaweeds is relatively stable, highly soluble, and exhibits strong absorption in the visible light spectrum. Three major categories of phycobiliproteins (phycocyanins, allophycocyanins, and phycoerythrins) are the major light-harvesting pigments in red seaweeds and are regularly used as fluorescent probes in scientific experiments (104).

### 3.5. Peptides derived from seaweeds

Marine bioactive peptides are typically composed of approximately 3–40 amino acids, and their activities are determined by their amino acid sequences and composition. These amino acids are not active within the sequence of the parent protein but can be released during gastrointestinal digestion, food processing, and fermentation. Aspartic and glutamic acids are the predominant amino acids found in seaweeds. Recently, seaweed-derived peptides have been widely investigated in the nutraceutical and pharmaceutical industries because of their health benefits. Seaweed-derived bioactive peptides were detected to be involved in various biological functions, including antioxidant (105, 106), anticancer (107, 108), antihypertensive (109), and anti-atherosclerotic effects (110). Bioactive peptides are believed to positively affect the body’s ability to function and thus influence human health (111–113).

## 4. Impact of seaweed-derived components on metabolic syndrome via regulation of gut microbiota or organic acid

### 4.1. Obesity and hyperlipidemia

The dietary intake of seaweeds or seaweed-derived bioactive molecules has been shown to reduce the prevalence of (or alleviate) certain chronic diseases, including obesity and obesity-induced hyperlipidemia. Representative studies related to the potential of seaweed-derived bioactive compounds in relieving obesity via modulation of gut microbiota are listed in Table 2.

**Table 2:** Effects of seaweed-derived compounds on obesity or hyperlipidemia through gut microbiota regulation.

Compounds (Source)	Model and dose	Significant observations and the related mechanisms	Alterations of the gut microbiota	Generated SCFAs	Ref.
Fucoidan (−)	Obese mice; 50 or 250 mg/kg/d	↓BW, BMI, TCHO, TG, LDL, LPS, TNF-α, and total bile acid; ↑HDL, organ index, liver steatosis, small intestine structure.	↑*Faecalibacterium prausnitzii*	N/A	(114)
Fucoidan (*Saccharina japonica*)	Obese mice; 0.6 mg/ml	↓BW, epididymal fat, adipocyte size and number, lipid deposition, colon muscle thickening; ↑Impaired glucose, lipid metabolism.	↑*Bacteroides sartorii*, *Bacteroides acidifaciens*	N/A	(115)
Fucoidan (*Undaria pinnatifida*)	Obese mice; 50 or 100 mg/kg/d	↓Serum TCHO, LDL-C, liver cholesterol levels; ↓Cholesterol-related proteins (HMGCR, SREBP-2) in liver.	↑Bacteroidetes, *Alloprevotella*; ↓Firmicutes, *Staphylococcus*, *Streptococcus*.	N/A	(116)
Fucoidan (*Laminaria japonica*, *Ascophyllum nodosum*)	Obese mice; 200 mg/kg	↓BWG, fat mass, serum lipid profile, hepatic steatosis, metabolic endotoxemia and systematic inflammation; ↑Glucose intolerance and insulin resistance.	↑*Akkermansia muciniphila*, *Clostridiales vadinBB60*, *Alloprevotella*, *Blautia*, *Bacteroides*.	N/A	(117)
Fucoidan (*Laminaria japonica*)	Obese mice; 100 and 200 mg/kg/d	↓BW, energy intake, liver oxidative stress and inflammation, hepatocyte steatosis; ↑Insulin resistance, dyslipidemia, hepatorenal function; ↓PI3K-Akt–mTOR axis, SREBP-1c/PPARγ.	↑*Verrucomicrobia*, *Akkermansia muciniphila*.	↑Acetic acid, propionic acid, butyric acid, isobutyric acid, valeric acid, isobutyric acid.	(118)
Fucoidan (*Laminaria japonica*)	Obese mice; 0.25% solution as drinking water	↓BWG, body fat mass, epididymal adipose tissue weight, lipid deposition, liver MDA level, fecal carbohydrate levels; ↑Lean mass, intestinal morphology, lipid profile, nutrient utilization, fecal fat levels; ↑GPR41/GPR43.	↑*Rikenellaceae*, *Bacteroidales* S24_7; ↓*Pseudomonas* spp., *Lachnoclostridium* spp.	↑Acetic acid and butyric acid in fecal contents; ↓Acetic acid in cecal contents.	(119)
Fucoidan (*Sargassum fusiforme*)	Obese mice; 200 mg/kg/d	↓BWG, BMI, abdominal fat, adipose size/numbers, serum TCHO, TG, LDL-C, FFA, ALT, and AST, MDA, 4-HNE-modified protein; ↑Insulin resistance, CAT activity, GSH/GSSG ratio, Nrf2, intestinal integrity and inflammation; ↓Il-6, *Tnf-α*, and *Il-1β*; ↑Zo-1, Occludin-1.	↑Bacteroidetes, *Bacteroides*, *Lactobacillus*, *Alistipes*, p_*Bacteroidetes*, o_*Bacteroidales*, g_*Alistipes*, s_*Alistipes_shahii*, g_*Parabacteroides*, s_*Parabacteroides_goldsteinii*, g_*dubosiella*, s_*Faecalibacterium_prausnitzi*, s_*Clostridium_sp*_A5F502; ↓Firmicutes.	N/A	(120)
Fucoidan (*Saccharina japonica*)	Obese mice; 1 mg/ml	↓D-lactate dehydrogenase and α-galactosidase coding genes; ↑FBG, cellulase-coding genes.	↑g_*Ruminococcus*, f_*Ruminococcaceae*.g_, g_*Akkermansia*, g_*Bacteroides*; ↓Bacteroidetes, Firmicutes, g_*Oscillospira*.	N/A	(121)
Fucoidan (*Sargassum fusiforme*)	Obese mice; 200 mg/kg/d	↓FBG, IR index, MDA level, ceramide levels in serum and colonic; ↑Phosphorylation of Akt, GSH/GSSG ratio, TUDCA levels; ↓4-HNE-modified protein, FXR/SHP signaling, SPT/CerS expression; ↑Nrf2 signaling.	↑*Clostridium*; ↓*Lachnospiraceae*.	N/A	(122)
Fucoidan (*Undaria pinnatifida*)	Obese mice; 100, 300, and 500 mg/kg/d	↓BWG, spleen weight, hepatic lipid droplets infiltration, adipocytes sizes, FBG, TC, TG, LDL-C, MDA levels, soluble carbohydrates in feces; ↑Cecum weight, distribution of fat mass and lean mass, serum HDL-C, SOD levels, intestinal permeability and inflammation, lipid levels in feces.	↑Bacteroidetes, *Lactobacillus*, *Bacteroidales*_S24-7, *Prevotellaceae*_UCG-001; ↓Firmicutes, *Desulfovibrionales*, *Clostridia*, *Lachnospiraceae*, *Ruminococcaceae*, *Desulfovibrio*.	↑Acetate, propionate, butyrate.	(123)
Alginate (*Laminaria japonica*)	Obese mice; 400 mg/kg/d	↓Hyperlipidemia, insulin resistance, glucose tolerance, endotoxemia-induced inflammation; ↑Intestinal barrier integrity; ↓ZO-1 and occludin protein levels.	↑Bacteroidetes, Actinobacteria, *Lactobacillus*, *Akkermansia genera*; ↓Proteobacteria, *Morganella*, *Proteus*, *Providencia*, *Enterobacteriaceae*.	N/A	(124)
Alginate (*Laminaria japonica*)	Obese mice; 0.3% solution as drinking water	↓BWG, fat accumulation, serum TC, TG, and LDL-C; ↑HDL-C levels.	↓*Clostridia, Deltaproteobacteria, Deferribacteres*; ↑*Bacteroidia*.	↑Production of SCFAs.	(125)
Alginate (−)	Obese mice; 5% formulation	↓TG, LDL-C, FBG, serum insulin, Fat accumulation, inflammation in the liver; ↓Lipogenesis marker mRNA expression levels, inflammatory cytokines, serum endotoxin levels.	↑*Akkermansia muciniphila, Lactobacillus reuteri, Lactobacillus gasseri, Bacteroides acidifaciens*; ↓*Deferribacteres, Erysipelotrichaceae, Lachnospiraceae, Rikenellaceae, Streptococcaceae*.	↑Acetic acid, propionic acid, butyric acid, isobutyric acid, pentanoic acid, isopentanoic acid.	(126)
Laminarin (−)	Obese mice; 1% solution as drinking water	↓BWG; ↑Carbohydrate-active enzymes (glycoside hydrolases and polysaccharide lyases).	↓Firmicutes; ↑Bacteroidetes, *Clostridium cluster XIVa*, *Parabacteroides*, *Bacteroides*, *Clostridium*_*XIVb*, *Clostridium*_*XI*.	N/A	(127)
Carrageenan (*Kappaphycus alvarezii*)	Obese mice; 5%, w/w	↓BWG, Lee’s obesity index, total fat percentage, liver weight, adipocytes size; ↑Serum biochemical profile.	↑Bacteroides-to-Firmicutes ratio, *Prevotellaceae, Alistipes, Parasutterella, Alloprevotella, Oscillibacter, Melainabacteria, Butyricimonas*; ↓*Lactobacillus* sp., *Clostridia, Erysipelotrichaceae, Blautia, Lachnospiraceae*.	↑Total SCFAs	(128)
Carrageenan (−)	Obese mice; 0.2 and 1% formulation	↓BW, body fat, FBG, serum TC, TG, LDL-C, TNF-α, LPS levels; ↑Intestinal barrier function damage.	↓Firmicutes, Proteobacteria, *Lachnospiraceae*, *Desulfovibrionaceae*; ↑Bacteroidetes, *Cyanobacteria*.	↑Butyric acid	(129)
Carrageenan (*Sarconema filiforme*)	Obese rats; 5% *S. filiforme* (*w/w*)	↓BW, fat mass and deposition in liver, systolic blood pressure, cardiac infiltration of inflammatory cells, collagen deposition, plasma TCHO, TG; ↑Diastolic stiffness.	No effect on Firmicutes to Bacteroidetes ratios.	N/A	(130)
Rhamnan sulfate (*Monostroma nitidum*)	1. Obese mice; 0.25 mg/g 2. Human clinial trial; 100 mg/day	↓Plasma lipids; ↑Fecal volume, calorie excretion, frequency of dejection.	↑Bacteroidetes, *Negativicutes*, *Acidaminococcales*, and *Veillonellales*; ↓Firmicutes, *Clostridia*.	N/A	(131)
Fucoxanthin (*Undaria pinnatifida*)	Obese mice; 0.05 and 0.1% in diet	↓BWG, FBG, insulin, HOMA-IR value, serum LPS, hepatic steatosis, adipocyte hypertrophy; ↑HDL-C level; ↓Ileum IL-6 and TNF-α levels; ↑↓Ileum IL-10 levels.	↓Firmicutes, *Faecalibaculum*, *Lachnoclostridium*, *Lachnospiraceae*_NK4A136_group; ↑Bacteroidetes, *Bacteroidales*_S24-7_group, *Bifidobacteriaceae*, *Enterococcaceae*, *Ruminococcaceae*, *Anaerotruncus*, *Romboutsia*, *Streptococcus*, *Enterococcus durans*, *Lactococcus lactis*, *L. gasseri*, *Lactobacillus equicursoris*, *Lactobacillus helveticus*, *Lactococcus raffinolactis*.	↑Butyrate	(132)

N/A, not analyzed; HFD, high-fat diet; BW, body weight; BWG, body weight gain; BMI, body mass index; TCHO, total cholesterol; TG, triglyceride; LDL, low-density lipoprotein; HDL, high-density lipoprotein; LPS, lipopolysaccharide; FBG, fasting blood glucose; HOMA-IR, homeostasis model assessment of insulin resistance; HMGCR, 3-hydroxy-3-methylglutaryl coenzyme A reductase; SREBP-2, sterol regulatory element-binding protein 2; FFA, free fatty acid; SCFA, short-chain fatty acid; MDA, malondialdehyde; CAT, catalase, ALT, alanine aminotransferase; AST, aspartate aminotransferase; GSH, glutathione; GSSG, oxidized glutathione; TUDCA, tauroursodeoxycholic acid; ROS, reactive oxygen species; STZ, streptozotocin; NOD, non-obese diabetic mouse; ZO-1, zonula occludens-1; IL-6, Interleukin-6; TNF-α, tumor necrosis factor-α.

#### 4.1.1. Polysaccharides against obesity via modulating gut microbiota

*In vivo* studies have revealed the benefits of fucoidan in a high-fat diet (HFD)-induced obesity mouse model, including a reduction in body weight gain or BMI, improved lipid profiles in the serum and liver, decreased lipid deposition in adipocytes and hepatocytes, and suppressed adipocyte hypertrophy (Table 2). The mechanisms of action of fucoidans in the prevention and treatment of obesity include decreased energy intake, regulation of lipid metabolism by affecting lipid absorption, and improved anti-oxidant capacity (99). In addition, the gut microbiota has attracted attention as a crucial mechanism involved in the fucoidan-induced alleviation of obesity.

The gut microbiota is mainly composed of two dominant bacterial phyla, Firmicutes and Bacteroidetes, which represent 90% of gut bacteria (133). Firmicutes contain numerous carbohydrate metabolism enzymes that contribute to macronutrient metabolism and allow greater energy absorption (134), whereas Bacteroidetes are responsible for the degradation of many complex glycans. Therefore, Bacteroidetes is positively correlated with obesity, but Firmicutes is negatively correlated, and previous studies have found that obese individuals have an increased Firmicutes/Bacteroidetes ratio (135). Fucoidan administration to HFD-induced obese mice resulted in a reversed Firmicutes/Bacteroidetes ratio compared with that in HFD-fed mice (116, 120, 121, 123). Therefore, the anti-obesity effect of fucoidan may be partially related to alterations in the Firmicutes/Bacteroidetes ratio. At the genus level, fucoidan improves the bacterial proportions of *Lactobacillus*, *Faecalibacterium*, *Blautia*, *Bacteroides*, *Alistipes*, *Ruminococcus*, and *Alloprevotella*, which are beneficial bacteria negatively correlated with obesity (116, 117, 120, 121, 123). In addition, the abundances of Firmicutes, *Staphylococcus*, *Streptococcus*, *Pseudomonas* spp., *Lachnoclostridium* spp., *g_Oscillospira*, *Lachnospiraceae*, *Desulfovibrionales*, and *Clostridia*, which are positively correlated with obesity, were significantly decreased by fucoidan administration in HFD mice (114, 116, 119, 121, 123). A notable bacterial genus, *Akkermansia*, has been reported to be negatively correlated with obesity (136). Oral administration of *Akkermansia* reverses HFD-induced metabolic syndrome, indicating its potential use for treatment by modulating the gut microbiota (137). Several studies have reported that fucoidan has powerful effects on increasing the abundance of *Akkermansia*, which may also partly explain the beneficial effect of fucoidan in modulating this gut microbe (117, 118, 121). In addition, fucoidan alleviates gut dysbiosis, consequently improving nutritional utilization by decreasing total fecal carbohydrates and increasing fecal fat levels (119, 123). The intestinal contents of HFD-fed mice showed that fucoidan treatment significantly increased SCFAs such as acetic acid, propionic acid, butyric acid, isobutyric acid, valeric acid, and isobutyric acid (118, 119, 123), which greatly contributed to the control of body weight, glucose homeostasis, and insulin sensitivity (22). Notably, fucoidan treatment ameliorates HFD-induced intestinal structural damage, including thickened colon muscle tissue, decreased cecal weight, shortened colon length, and impaired colon mucosa structure (115, 119, 123). This therapeutic effect may be caused by regulated beneficial bacteria (*Bacteroides*, *Lactobacillus*, and *Akkermansia*) that are positively related to intestinal integrity and inflammation (120).

Alginate also has a strong effect on the gut microbiota of HFD-induced obese mice. Li et al. reported that HFD-induced obese mice fed unsaturated alginate oligosaccharides (UAOS; 400 mg/kg/day) for 7 weeks showed attenuated obesity-related metabolic abnormalities, such as hyperlipidemia, insulin resistance, and low-grade inflammation, via modulation of the gut microbiota (124). UAOS treatment partially reversed HFD-induced gut dysbiosis by increasing the abundance of Bacteroidetes and Actinobacteria and decreasing the abundance of Proteobacteria. In contrast, the abundance of *Morganella*, *Proteus*, *Providencia*, and *Enterobacteriaceae* was significantly reduced. Interestingly, UAOS treatment selectively increased the abundance of beneficial intestinal bacteria (*Lactobacillus* and *Akkermansia*) and decreased the abundance of inflammatory bacteria (*Parabacteroides*). Another study involving HFD-induced obese mice showed that low-molecular-weight alginate improved body weight gain, fat accumulation, and hyperlipidemia by modulating the gut microbiota, increasing the number of beneficial bacteria (*Bacteroides*), and decreasing the number of harmful bacteria (*Lachnospiraceae*) in the gut of alginate-treated obese mice (125). The increased production of SCFAs showed that low-molecular-weight alginates benefit host health through SCFA-mediated pathways. Wang et al. also demonstrated that the supplementation of HFD-induced obese mice with alginate oligosaccharides significantly ameliorated metabolic disorders by promoting the growth of *Akkermansia muciniphila*, *Lactobacillus reuteri*, and *Lactobacillus gasseri*. These bacteria exhibit multiple correlations with metabolic traits, including negative correlations with total cholesterol (TC), LDL-C, triglyceride (TG) levels, fasting blood glucose (FBG), and serum endotoxins (126).

#### 4.1.2. Other anti-obesity seaweed-derived compounds via modulation of gut microbiota

The anti-obesity effects of other bioactive compounds derived from seaweeds, such as laminarin, carrageenan, porphyran, rhamnan sulfate, fucoxanthin, and bromophenol, are also related to gut microbiota modulation. Nguyen et al. found that laminarin treatment in HFD mice decreased body weight gain with an improved Firmicutes/Bacteroidetes ratio, enriched beneficial bacteria (*Clostridium cluster XIVa*, *Parabacteroides,* and *Bacteroides*), and reduced the abundance of potentially pathogenic bacteria (*Clostridium*_XIVb and *Clostridium*_XI). A high abundance of carbohydrate-active enzymes (glycoside hydrolases and polysaccharide lyases) has also been reported (127).

Two studies found that κ-carrageenan relieved HFD-induced body weight gain, hyperlipidemia, and body fat accumulation in mice (128, 129). Gut microbiota profiling analysis revealed that the Firmicutes to Proteobacteria ratio was restored compared to that in obese mice. There was an increased abundance of Prevotellaceae and the genera *Alistipes* and Bacteroidetes, which negatively correlated with hepatic and serum lipid profiles, and a decreased abundance of *Blautia*, *Lachnospiraceae*, and *Erysipelotrichaceae*, which positively correlated with body weight, FBG, and serum lipid profiles.

RS was also found to regulate the gut microbiota during the treatment of metabolic syndrome. Shimada et al. reported that RS administration in HFD-fed mice significantly increased fecal volume and calorie excretion (131). The latter authors further performed a clinical trial in which RS (100 mg/day) was administered to subjects with infrequent defecation (3–5 times/week) in a double-blind, placebo-controlled manner. After 2 weeks of consumption, the subjects showed increased dejection frequency without changes in body weight and blood lipid levels. Furthermore, the gut microbiota exhibited an increased Bacteroidetes-to-Firmicutes ratio. Decreased proportions of *Clostridia* and increased proportions of *Negativicutes*, *Acidaminococcales,* and *Veillonellales* were observed. *Clostridia* are positively associated with constipation by producing medium-length fatty acids that increase water absorption and dry feces (138). Additionally, increased *Negativicutes* and *Acidaminococcales* have been found to be positively related to the alleviation of constipation, and *Negativicutes* increase during *Bifidobacterium*-based probiotic treatment for constipation (139). The main metabolite of *Acidaminococcus* is acetic acid, which promotes intestinal peristalsis and relieves constipation (140).

Overall, the bioactive compounds of seaweeds described above may comprise promising anti-obesity agents that act via modulation of the gut microbiota. Although PUFAs (141), phlorotannin (79), agar (142), and ulvan (143) have anti-metabolic syndrome functions, few studies have investigated their effects on the gut microbiota.

### 4.2. Diabetes

It is well known that people with metabolic syndrome have a significantly higher risk of developing type 2 diabetes mellitus (T2DM), which is associated with insulin resistance, of which obesity is a major contributor. Previous studies have shown a significant effect of using seaweeds to treat diabetes, which is achieved by regulating the gut microbiota. The representative studies are listed in Table 3.

**Table 3:** Effects of seaweed-derived compounds on diabetes through gut microbiota regulation.

Compounds (Source)	Model and dose	Significant observations and the related mechanisms	Alterations of the gut microbiota	Generated SCFAs	Ref.
Fucoidan (*Sargassum fusiforme*)	Diabetic mice; 100 mg/kg/d	↓FBG, food and water intake, hyperlipidemia, epididymal fat deposition, oxidative stress; ↑Glucose tolerance, pathological changes in the heart and liver tissues, (R)-carnitine and choline levels in the colon.	↑Bacteroides, *Faecalibacterium*, *Blautia*, *Ruminiclostridium*, *Parabacteroides goldsteinii*, *Enterococcus faecalis*, *Lactococcus lactis*; ↓*Desulfovibrio*.	N/A	(144)
Fucoidan (*Sargassum fusiforme*)	Diabetic mice; 100 mg/kg/d	↓FBG, dietary intake, water intake, oxidative stress; ↑Pathological changes in the heart and liver, liver function.	↑Bacteroidetes to Firmicutes ratio, *Tidjanibacter massiliensis*, *Streptococcus danieliae, Aerococcus viridans, Alloprevotella, Alistipes, Odoribacter, Millionella, Roseburia, Erysipelatoclostridium, Aerococcus, Rikenella, Lachnoclostridium, Acetatifactor*; ↓*Harryflintia, Desulfovibrio*, *Bacteroides*, *Faecalibaculum*, *Anaerotruncus*, *Blautia*, *Ruminiclostridium*, *Mucispirillum*, *Parabacteroides*, *Oscillibacter*, *Bilophila*, *Butyricimonas*, *Candidatus*, *Romboutsia*, *Dubosiella*.	N/A	(145)
Fucoidan (*Fucus vesiculosus*)	NOD mice; 300 and 600 mg/kg/d	↑Serum insulin level, Th2-bias ed. cytokine response; ↓Onset and the development of diabetes, Th1 pro-inflammatory cytokines levels, MHC class II and CD86, TLR4 and downstream molecules’ expression in the pancreas.	↓*Bacteroidaceae*, *Prevotellaceae*, *Alloprevotella*, *Enterorhabdus*, *Mucispirillum*; ↑*Lactobacillus*, *Akkermansia*; *Lactobacillaceae*, *Clostridium* XlVa, *Anaerofustis*.	N/A	(146)
Carrageenan (−)	Diabetic rats; 270 mg/kg/d	No effects on glycemic control, serum TC, TG, and LDL-C; ↑Serum GLP-1; ↓Serum leptin levels.	↑Bacteroidetes; ↓Actinobacteria.	↓Colonic acetic acid	(147)
Porphyran (*Pyropia yezoensis*)	High-sucrose-fed Drosophila melanogaster; 15, 25, and 50 g/kg	↓TG, circulating sugars contents.	↓*Escherichia–Shigella, Fusobacterium*; ↑*Bacillus, Akkermansia*.	N/A	(148)
Bromophenol (*Rhodomela confervoides*)	BKS db diabetic mice; 100 mg/BW/d	↓FBG, water intake; ↑Metabolic disorder; ↑Propanoate metabolism; ↓Starch, sucrose, amino sugar, nucleotide sugar metabolism.	↑*Lachnospiraceae*, *Bacteroides*, *Akkermansia*.	N/A	(149)

TLR4, Toll-like receptor 4; GLP-1, Glucagon-like peptide-1.

#### 4.2.1. Effect of polysaccharides against diabetes via gut microbiota

In HFD and streptozotocin (STZ)-induced T2DM mouse experiments, fucoidan effectively decreased FBG levels and improved glucose tolerance (122, 144, 145). The potential mechanism underlying the amelioration of T2DM by fucoidan is thought to involve the enrichment of benign microbes (*Bacteroides*, *Faecalibacterium,* and *Blautia*) and a decrease in the abundance of Proteobacteria (especially *Desulfovibrio*). *Desulfovibrio* is a sulfate-reducing bacterium and opportunistic pathogen associated with inflammatory diseases (150). Wu et al. (144) further analyzed the correlation between *Desulfovibrio* and metabolic parameters and found that *Desulfovibrio* was positively correlated with FBG levels and that the decreased abundance of *Desulfovibrio* by fucoidan may contribute to improving glucose metabolism. Fucoidan treatment was also found to increase SCFA production and promote a relatively high abundance of SCFA-producing bacteria such as *Blautia*, *Faecalibacterium*, and *Alloprevotella*. UAOS also improved obesity-induced insulin resistance and increased serum insulin levels in diabetic mice (124, 126). UAOS treatment significantly increases the abundance of *Acetatifactor*, which is positively associated with glucose-insulin homeostasis (124). *Anaerotruncus*, *Tyzzerella*, *Acetatifactor*, and *Intestinimonas* were decreased by alginate oligosaccharide, and their reductions showed positive and negative relationships with FBG and serum insulin, respectively (126). The growth of *Akkermansia. muciniphila* also improved, showing a positive correlation with serum insulin (151). These results suggest the potential use of alginate oligosaccharides as novel prebiotic agents for the treatment of diabetes and related metabolic diseases.

Wang et al. reported that dietary supplementation of κ-carrageenan in HFD-induced obese mice significantly decreased FBG levels and effectively increased glucose tolerance (129). The relative abundances of Firmicutes, Proteobacteria, *Lachnospiraceae*, and *Desulfovibrionaceae* were decreased by κ-carrageenan, and these microbes showed a positive relationship with FBG. However, a recent study found that carrageenan did not affect glycemic control in HFD- and STZ-induced type 2 diabetic rat models (147). Porphyrans have also been found to regulate the gut microbiota during the treatment of metabolic syndrome. He et al. found that *Pyropia yezoensis* porphyran ameliorated the circulating sugar content in high-sucrose-fed *Drosophila melanogaster* larvae (148). The abundance of bacteria causing metabolic abnormalities (*Escherichia, Shigella*, and *Fusobacterium*) was decreased, whereas the abundance of beneficial bacteria (*Bacillus* and *Akkermansia*) increased. However, further mammalian experiments and clinical trials are needed to confirm the effects of carrageenan and porphyran against diabetes by modulating the gut microbiota.

#### 4.2.2. Effect of polyphenols against diabetes via alteration of gut microbiota

Recently, a natural bromophenol (3-bromo-4,5-bis(2,3-dibromo-4,5-dihydroxybenzyl)-1,2-benzenediol; BDB) isolated from the marine red algae, *Rhodomela confervoides,* was found to alleviate T2DM in diabetic BKS db mice (149). The antidiabetic effect of BDB is closely related to the modulating structure of the gut microbiota, including the increased abundance of short-chain fatty acid (SCFA)-producing bacteria *Lachnospiraceae* and *Bacteroides* and the elevation of *Akkermansia* spp. to enhance glucose homeostasis. Interestingly, BDB alleviated metabolic disorders in T2DM mice by promoting propanoate metabolism and inhibiting starch, sucrose, amino sugars, and nucleotide sugar metabolism.

### 4.3. Cancer

Although cancer has traditionally been considered a growth disorder, recent evidence suggests that it should be considered a metabolic disease. When tumors grow, they alter their metabolic programs to meet and even exceed the bioenergetic and biosynthetic demands of continued abnormal cell growth. The cancer-promoting effects of gut microbiota dysbiosis have been extensively investigated in recent years (152). In addition, the gut microbiota has been linked to cancer and has been shown to modulate the effects of anticancer drugs.

We found fucoidan to be the most studied seaweed, having been the focus of investigations since the 1980s. It has been found to both decrease the viability of a wide range of cancer cell lines *in vitro* and to inhibit tumor growth and metastasis *in vivo* (153). Ten studies have elucidated the anticancer effects of fucoidan by regulating gut microecology. Xue et al. investigated the effects of fucoidans derived from *Fucus vesiculosus* in a 1,2-dimethylhydrazine-induced colorectal cancer rat model (154). Dietary fucoidan significantly reduces tumor incidence, decreases tumor weight, and increases tumor cell apoptosis. 16S rDNA high-throughput sequencing revealed that gut microbial dysbiosis in the cancer group resulted in an increased Bacteroidetes/Firmicutes ratio. However, fucoidan administration ameliorated this disorder, decreased the abundance of *Prevotella* (an opportunistic pathogen), and increased the abundance of *Alloprevotella* (an anti-inflammatory bacterium). The production of SCFAs (especially butyric and caproic acids) increased significantly in the fucoidan-treated groups. Another previous study demonstrated the potential of fucoidan as a gut microbiota modulator for breast cancer prevention (155). Oral administration of fucoidan to breast cancer model rats significantly ameliorated the damaged mucosal morphology of the jejunal tissue by enhancing the expression of tight junction proteins, including ZO-1, occludin, Claudin-1, and Claudin-8. Interestingly, the Bacteroidetes/Firmicutes ratio increased in the intestines of fucoidan-treated cancer model rats. In this example, the abundance of *Prevotella* increased, which may have contributed to increased SCFAs concentrations. Additionally, fucoidan decreased the abundance of Sutterella, which produces endotoxins that are harmful to animals. Therefore, fucoidan may prevent tumorigenesis or suppress tumor growth by regulating gut microecology. However, more evidence (such as that from clinical trials) is needed to further elucidate the relationship between fucoidan and different types of cancers via gut microbiota regulation.

Alginate has shown considerable activity against murine tumors, such as sarcoma 180 (156); however, studies on the relationship between the antitumor activity of alginate and gut microbiota are lacking. Instead, we found two studies that examined the effects of alginate oligosaccharide (AOS) on the adverse effects of chemotherapy with anticancer drugs such as busulfan. Zhang et al. (157) reported that fecal microbiota transplantation from AOS-treated mice to busulfan-treated ICR mice abolished busulfan-induced small intestinal mucositis and gut microbiota dysbiosis. The ratio of Bacteroidetes/Firmicutes improved, and the abundance of beneficial microbes, such as *Leuconostocaceae* and *Lactobacillales*, increased to assist in blood metabolome recovery. In another study, AOS increased murine sperm concentration and motility, and rescued busulfan-disrupted spermatogenesis (132). These results indicate that alginate can be used to prevent small intestinal mucositis and improve fertility in patients undergoing chemotherapy.

### 4.4. Hypertension

Hypertension is a common disease associated with a high risk of cardiovascular mortality, and often occurs in tandem with metabolic disturbances, specifically dyslipidemia (158). Sodium alginate oligosaccharides have been reported to attenuate several types of hypertension in rat models, such as Dahl salt-sensitive hypertension, spontaneous hypertension, and pulmonary hypertension (159–162). However, compared with the number of reports on the use of seaweed and seaweed-derived components against hypertension, few studies have investigated the relationship between these components and the related gut microbiota. Han et al. found that the oral administration of potassium alginate oligosaccharides (PAO) significantly decreased systolic blood pressure and mean arterial pressure in spontaneously hypertensive rats (163). The relative abundances of *Phascolarctobacterium* and *Prevotella_9* were markedly decreased to nearly zero by PAO and were positively correlated with systolic blood pressure. In addition, lactic acid levels increased with a decrease in the abundance of *Lactobacillus*, which may be correlated with changes in systolic blood pressure. Carrageenans from the red seaweed *Sarconema filiforme* were also found to decrease high systolic blood pressure induced by HFD in rats (130), while there was an increase in the relative abundance of the family *Ruminococcaceae UCG-014* (belonging to the phylum Firmicutes), which was positively correlated with systolic blood pressure.

### 4.5. Immune disorders

Any immune system disorder causes the body’s defense mechanisms to malfunction or become disabled. Immune disorders are characterized by inflammatory symptoms. Several studies have investigated the immunomodulatory and regulatory effects of bioactive seaweed-derived compounds (164). Administration of fucoidan extracted from Okinawa mozuku (*Cladosiphon okamuranus*) to healthy adult zebrafish significantly altered the gut microbiota composition by increasing the relative abundance of *Comamonadaceae* (AB076847) and *Rhizobiaceae* (*Shinella granuli*), which was accompanied by decreased expression levels of the pro-inflammatory cytokine *il1b* (165). Tang et al. investigated the effects of fucoidan obtained from *Laminaria japonica* on the immune response and intestinal microflora in a mouse model of immunosuppressant-induced immune disorder (166). Fucoidan treatment significantly enhanced the spleen and thymus indices, relieved the cellular immune response by promoting splenic lymphocyte proliferation, and ameliorated immunosuppression in mice by enhancing cytokine and IgG production. The regulated gut microflora composition included a remarkably decreased abundance of *Lactobacillaceae*, *Bacilli*, and *Lactobacillus,* which was positively correlated with pro-inflammatory cytokine levels, such as IL-6 and TNF-α. Moreover, the abundance of *Alistipes* significantly increased, which was negatively correlated with MDA levels and positively correlated with SOD and GSH-Px activities. In addition, the disturbance in five gut microbiota species, *Erysipelotrichia*, *Turicibacter*, *Romboutsia*, *Peptostreptococcaceae*, and *Faecalibaculum*, was significantly reversed by fucoidan treatment. Another previous study revealed that fucoidan treatment in a mouse model of psoriasis (a chronic autoimmune inflammatory disease) significantly alleviated symptoms and decreased facial scratching (167). Analysis of fecal microbiota revealed that fucoidan increased the relative abundance of Bacteroidetes and decreased that of Firmicutes. Two *Clostridiales* families, *Lachnospiraceae* and *Ruminococcaceae*, were reduced, which are related to pro-inflammatory cytokine secretion and autoimmune disorder induction. The increased relative abundance of *Desulfovibrionaceae* and *Bacteroidetes acidifaciens* induced by fucoidan may be associated with the anti-inflammatory effect noted in the improvements in psoriasis symptoms and may promote the expression of secreted IgA in the large intestine to rearrange the intestinal environment by regulating the immune response. Therefore, fucoidan has the potential to be used as an immunoregulatory adjuvant to improve immunosuppression.

The intestinal tract is the major organ involved in immune and inflammatory reactions, and the gut microbiota adversely affected by metabolic syndrome can be reversed using seaweed and seaweed-derived components, such as fucoidan, alginate, and fucoxanthin (117, 118, 120, 126, 168). The enhanced abundance of beneficial bacteria (e.g., *Lactobacillaceae* and *Roseburia*) may contribute to the immunoregulatory effects of these bioactive compounds. Fucoxanthin was found to reduce the secretion of pro-inflammatory cytokines (TNF-α and IL-6) and increase IL-10 levels in the ileum of HFD-induced obese mice owing to the decreased abundance of *Faecalibaculum*, which was proven to be positively correlated with LPS and TNF-α and negatively correlated with IL-10 (132). Although we found no publications focusing on the effects of ulvan on immune disorders via the regulation of the gut microbiota, an *in vivo* study revealed that feeding ulvan extracted from *Ulva ohnoi* to normal mice for 28 days positively modulated the gut microbiota (169). The bacterial classes *Bacteroidia*, *Bacilli*, *Clostridia*, and *Verrucomicrobia*, which are probiotics that assist in maintaining the intestinal barrier, were significantly increased in the fecal samples of ulvan-fed mice. An increased relative abundance of *Lachnospiraceae* (class *Clostridia*) has been observed, and reduced levels of these bacteria have been linked to inflammatory bowel disease and chronic gastrointestinal tract infections (170).

In summary, the bioactive compounds present in seaweeds may serve as functional molecules that regulate and modulate the human immune system. However, few such studies have been conducted to date, and further *in vivo* research and clinical trials are required to better understand their bioactivities.

### 4.6. Atherosclerosis and cardiovascular disease

Metabolic syndrome-induced atherogenic dyslipidemia, particularly hypertriglyceridemia and reduced HDL levels, is commonly associated with chronic inflammation and endothelial dysfunction, which subsequently accelerates atherosclerosis and increases cardiovascular risk (171). We found 11 publications that reported the anti-atherosclerosis or anti-cardiovascular disease effects of seaweed-derived components (nine and two publications, respectively). Of these, carrageenan appeared in three search results, but the focus of the associated papers was unrelated to the purpose of this study. Carrageenan was used to induce rodent-specific acute inflammation models, such as pleurisy or thrombosis; however, humans do not experience this phenomenon. Therefore, we did not discuss carrageenan further in this section.

Fucoidan from *Sargassum fusiforme* was reported to ameliorate pathological injury in the cardiac tissue of HFD- and STZ-induced mice, with an increase in enriched benign microbes, including *Bacteroides*, *Faecalibacterium,* and *Blautia* (144). The oral administration of PAO prevents heart failure and microbiome alterations in spontaneously hypertensive rats (163). There was an increase in microbial diversity and a decrease in the Firmicutes to Bacteroidetes ratio via reductions in the abundance of *Prevotella* and *Phascolarctobacterium*. The authors also found that *Prevotella* and *Phascolarctobacterium* were positively correlated with LPS, a mechanistic biomarker of cardiovascular disease (172). These findings indicate the potential of fucoidan and alginate in preventing the development of atherosclerosis or cardiovascular disease by improving gut dysbiosis.

### 4.7. Non-alcoholic fatty liver disease and non-alcoholic steatohepatitis

Non-alcoholic fatty liver disease results from the accumulation of fat in the liver, with two hallmarks of inflammation and liver damage (173). Although previous studies have found that seaweed-derived components reduce the incidence of NAFLD/NASH, only two previous studies focused on the role of gut microbiota. For example, Wang et al. reported that porphyran-derived oligosaccharides from *Porphyra yezoensis* administered to mice with HFD-induced NAFLD markedly reduced hepatic oxidative stress and lipid accumulation and relieved hepatic fibrosis (174). An increased abundance of *Akkermansia* and a decreased abundance of *Helicobacter* were observed. *Akkermansia* is considered to be a next-generation beneficial microbe for its effects on reversing obesity, inflammation, metabolic endotoxemia, and insulin resistance, whereas *Helicobacter* has been proven to be positively associated with NAFLD with respect to causing inflammation and hepatocyte ballooning. Another study revealed that treatment with the whole macroalga *Laminaria japonica* prevented HFD-induced NAFLD in a rat model (175). Although this study did not evaluate single compounds, consumption of whole seaweed ameliorated HFD-induced NAFLD by restoring gut microbiota dysbiosis.

## 5. Conclusion and future perspectives

**Figure 3:**
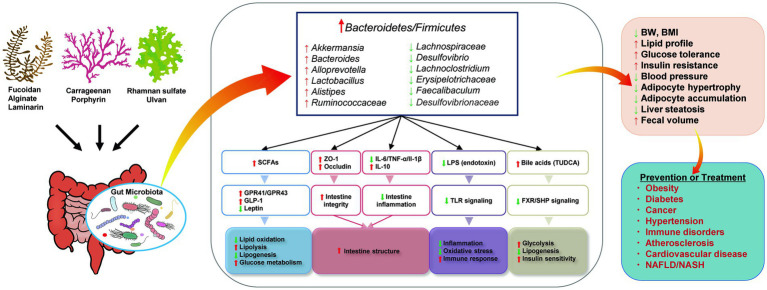
Proposed mechanisms for the effects of seaweed-derived bioactive components on improving metabolic syndrome via regulating gut microbiota. Illustrations were provided by Illust AC (https://www.ac-illust.com) with no copyright issue.

Studies have demonstrated that seaweeds and their bioactive components are potential foods that can be used to manage human metabolic syndrome, and that they have positive effects on obesity, hyperglycemia, hyperlipidemia, and hypertension. Animal experiments have shown that seaweed-derived bioactive components can decrease the risk of metabolic syndrome-related diseases by reducing the Firmicutes/Bacteroidetes ratio, increasing the relative abundance of beneficial bacteria, such as *Bacteroides*, *Akkermansia*, *Lactobacillus*, or decreasing the abundance of harmful bacteria, such as *Lachnospiraceae*, *Desulfovibrio*, *Lachnoclostridium*. The proposed mechanisms for the observed effects are summarized in Figure 3. Briefly, the regulated gut microbiota affects the generation of SCFAs and influences glucose and lipid metabolism. The intestinal structure can be improved by inhibiting intestinal inflammation and alleviating intestinal integrity. Altered microbiota inhibits endotoxin-induced inflammation and oxidative stress. Increased bile acid production may also be associated with improved glucose homeostasis and lipid metabolism. Thus, the interaction between the gut microbiota and seaweed-derived bioactive components plays a critical regulatory role in human health, and these compounds can be used as potential materials for drug development.

However, most of the published articles we collected focused on fucoidan and alginate, and very few papers have reported how other compounds affect gut microbiota and diseases. For example, in addition to fucoidan and alginate, other seaweed-derived compounds, such as rhamnan sulfate, have the potential to prevent or treat metabolic syndrome-related diseases by improving gut dysbiosis. Therefore, more *in vitro* and *in vivo* data are required to understand the functional roles and mechanisms of these components in balancing the gut microbiota and managing host health. In addition, only one clinical trial revealed a relationship between seaweed-derived biocomponents and modulated gut microbiota to alleviate metabolic syndrome-related diseases. However, further clinical trials are required to verify the therapeutic efficacy of these compounds. Although comparatively few studies have been conducted on whole algae, those that exist have shown promising results, such as prebiotic effects (176, 177). Therefore, whole seaweed consumption for the prevention and treatment of metabolic syndrome via the modulation of the gut microbiota needs to be investigated further. This reduces the economic and environmental costs of bioactive compound extraction.

## Author contributions

LZ, YS, MT, and NN: conceptualization. LZ: investigation. LZ and MB: writing—original draft preparation. LZ, YS, and MT: writing—review and editing. NN: supervision, project administration, and funding acquisition. All authors contributed to the article and approved the submitted version.

## Funding

This study received research grants from the chemical company Konan Chemical Manufacturing Co., Ltd.

## Conflict of interest

MT is an employee by Konan Chemical Manufacturing Co., Ltd. NN is a board member of Konan Chemical Manufacturing Co., Ltd.

The authors declare that this study received funding from Konan Chemical Manufacturing Co., Ltd. The funder had the following involvement in the study: manuscript preparation and decision to publish.

## Publisher’s note

All claims expressed in this article are solely those of the authors and do not necessarily represent those of their affiliated organizations, or those of the publisher, the editors and the reviewers. Any product that may be evaluated in this article, or claim that may be made by its manufacturer, is not guaranteed or endorsed by the publisher.
