# Supplementary material for: Beneficial effects of seaweed-derived components on metabolic syndrome via gut microbiota modulation
Source: Front Nutr. 2023 Jun 15;10:1173225. doi: 10.3389/fnut.2023.1173225 (PMC10311452; doi:10.3389/fnut.2023.1173225)
Supplement: Supplementary file 1 [file Data_Sheet_1.docx]

Supplementary Material

Beneficial Effects of Seaweed-derived Components on Metabolic Syndrome via Gut Microbiota Modulation

Liqing Zang ^*^, Maedeh Baharlooeian, Masahiro Terasawa, Yasuhito Shimada, Norihiro Nishimura

*** Correspondence:**

Liqing Zang
liqing@med.mie-u.ac.jp; Tel.: +81-59-233-5405

**Supplementary Figure S1.** Publication numbers were collected from the Web of Science based on a search using the keywords “seaweed-derived compounds,” “metabolic syndrome-related diseases,” and “Gut Microbiota (GM).”
